# Supplementary material for: A Comparative Study on the Lysosomal Cation Channel TMEM175 Using Automated Whole-Cell Patch-Clamp, Lysosomal Patch-Clamp, and Solid Supported Membrane-Based Electrophysiology: Functional Characterization and High-Throughput Screening Assay Development
Source: Int J Mol Sci. 2023 Aug 14;24(16):12788. doi: 10.3390/ijms241612788 (PMC10454728; doi:10.3390/ijms241612788)
Supplement: Supplementary file 1 [file ijms-24-12788-s001.zip › ijms-2488520-supplementary.pdf]

Supplementary Information

A comparative study on the lysosomal cation channel TMEM175 using automated whole-cell patch-clamp, lysosomal patch-clamp, and SSM-based electrophysiology: Functional characterization and HTS assay development.

Andre Bazzone[1*], Maria Barthmes[1], Cecilia George[1], Nina Brinkwirth[1], Rocco Zerlotti[1,2], Valentin Prinz[1], Kim Cole[1], Søren Friis[1], Alexander Dickson[3], Simon Rice[3], Jongwon Lim[4], May Fern Toh[4], Milad Mohammadi[5], Davide Pau[3], David J Stone[4], John J Renger[4], Niels Fertig[1*]

[1] Nanion Technologies, Ganghoferstr. 70a, 80339 Munich, Germany
[2] RIGeL - Regensburg International Graduate School of Life Sciences, Regensburg, Germany
[3] SB Drug Discovery, West of Scotland Science Park, G20 0XA Glasgow, Scotland
[4] Cerevel Therapeutics, 222 Jacobs St, Cambridge, Massachusetts, 02141, USA.
[5] Assay Works, Am BioPark 11, 93053 Regensburg, Germany
* Correspondence: A.B.: andre.bazzone@nanion.de; N.F.: niels.fertig@nanion.de

Table of Contents

Supplementary Methods: Experimental Procedures in SSME recordings

Supplementary Methods: Analysis procedures for SSME data

Figure S1: Western Blot

Figure S2: Ion selectivity of TMEM175 recorded with the SURFE²R 96SE

Figure S3: pH dependence of K^+ flux through TMEM175 recorded with the SURFE²R N1

Figure S4: HTS compatible workflow using the SURFE²R 96SE

Figure S5: Off-target compound effects recorded with the SURFE²R 96SE

Figure S6: Stability of electrophysiological recordings with the SURFE²R 96SE

Supplementary Methods: Experimental Procedures in SSME recordings

Measurement principles of SSME

Purified lysosomes are diluted in non-activating solution and sonicated using a tip sonicator to equilibrate the internal solution of the lysosomal membrane sample. The sample is then added to the SURFE²R sensor carrying the SSM. Sample membrane and SSM form a stable and capacitively coupled membrane system (Figure 1C) allowing for multiple fast solution exchange experiments on the same sensor using different ion compositions.

In SSME, a solution exchange from a non-activating solution (NA) to an activating solution (A) stimulates substrate translocation. The activating solution provides the substrate, while the non-activating solution usually contains equimolar concentrations of a non-substrate with similar physicochemical properties to prevent solution exchange artifacts.

For this research, two devices were used (Table 2): the single-well SURFE²R N1 platform with high flexibility for assay development (Figures 1D-G) and the SURFE²R 96SE platform that enables efficient drug screening using 96-sensor well-plates (Figures 1H-K). Assays have been developed on the SURFE²R N1 and subsequently transferred to the SURFE²R 96SE to enable a higher data throughput. While generally assays can be transferred between both instruments, workflow protocols are different. Most prominently, experiments on the SURFE²R N1 are carried out cumulatively on the same sensor, while measurements on the SURFE²R 96SE benefit from the parallelization and uses an in-well normalization procedure to perform automated cross-well analysis.

SSME recordings of TMEM175 on the SURFE²R N1

The SURFE²R N1 (Figure 1D) performs a solution exchange on a single sensor with 3 mm in diameter (Figure 1E) under continuous flow conditions at 200 µl/s with a maximum total volume of 2.5 ml per experiment. The currents are recorded using the SURFE²R N1 Control software (Figure 1F). Current traces usually last 3 seconds (Figure 1G). At the time point of 0.3 s the solution flow of non-activating solution starts, which generates a mechanical artifact. At 1.0 s the valve switches and the activating solution is injected, leading to the current rise corresponding to the TMEM175 channel activity when it reaches the sensor surface at ~1.1 s. K^+ translocation through TMEM175 charges the sensor; the increasing membrane voltage acts as a counter force to the K^+ concentration gradient and the capacitive current decays to baseline as a new electrochemical equilibrium is reached. The peak current of this on-signal is used as a measure for the macroscopic K^+ translocation rate through TMEM175. At 2.0 s the valve switches to the non-activating solution, which triggers K^+ efflux and generates an off-signal when the solution reaches the sensor surface at ~2.1 s. At the time point of 3.0 s the solution flow stops, leaving the sensor at initial conditions with 0 mV and 0 mM K^+. Subsequent solution exchange experiments may be performed using the same sensor. In SSME, only the on-signal is used for analysis.

Transferring the TMEM175 assay to higher throughput

The SURFE²R 96SE (Figure 1H) uses 96-sensor well-plates (Figure 1I) and performs 96 solution exchange experiments in parallel. SURFControl96 and DataControl96 software packages are used for automated recordings and data analysis (Figure 1J). Parallelization required significant changes in liquid handling, leading to a reduced duration of the current recording (Figure 1K), but also enables reduced solution consumption.

The SURFE²R 96SE carries 96 pipettes with a volume of 200 µl and solution exchange is performed by adding a stack of activating and non-activating solutions within the pipettes to a sensor well containing ~20-40 µl of non-activating solution at a speed of 200 µl/s. After the measurement the

liquid added during the measurement is removed and sensors are rinsed with non-activating solution to dilute the substrate and restore initial conditions. The SURFE^{2}R 96SE requires ~5 times less solution per measurement compared to the SURFE^{2}R N1 (Table 2). Despite the shorter measurement time window and due to the fast decay times of channel-like currents, on- and off-signals of TMEM175 are still easily separated (Figure 1K).

Supplementary Methods: analysis procedures for SSME data

The following process was applied to analyze most SURFE^{2}R 96SE datasets. Some variations apply, which are explained below. Most steps of the analysis procedure were automated using DataControl96.

1. **Measurements**
    a. Two 96-sensor well-plates are recorded, one containing the TMEM175 sample, the other containing the control sample; Recordings were performed on the same day, using the same measurement solution batch, measurement sequence and plate layout.
    b. All sensors are treated with a reference measurement, exchanging 50 mM Na^+ for 50 mM K^+ at pH 7.5 for normalization (REFERENCE), followed by a measurement with a different pH, ion species, or ion concentration (condition); in compound assays the recording is repeated in the presence of compound.
    c. Usually, 12 different conditions are applied, with N=8 sensors per condition, representing the sensors within one column of the 96-sensor well-plate.
2. **QC selection**
    a. Based on the QC settings outlined in the method section, sensor wells were removed from analysis.
3. **Determining and averaging artifact currents**
    a. Automated peak detection was set to extremum, to capture both negative or positive peak currents.
    b. Artifact peak currents from recordings on control samples were determined by subtracting the baseline current before the on-signal rise to correct for potential current off-sets.
    c. Then, artifact currents were averaged across sensors for each condition and the standard error of the mean ($SEM=SD/\sqrt{N}$) was calculated.
4. **Determining peak currents recorded with the TMEM175 sample**
    a. TMEM175 sample currents were determined by the same procedure as the artifact currents, without averaging across sensors.
5. **Subtracting artifact currents from TMEM175 sample currents**
    a. The peak current of each individual well containing the TMEM175 sample, is now adjusted by subtracting the average artifact current, which was recorded under the same condition.
    b. This procedure encompasses the correction of the REFERENCE recording for the corresponding artifact and the subtraction of the artifact present in the recording under the given condition, considering that the artifact may be influenced by the applied condition (for compounds, this includes off-target compound effects).
    c. The obtained TMEM175 net peak currents of each sensor-well now include a standard error defined by the artifact variation.

6. **In-well normalization**
    a. For each single well, we performed an in-well normalization: the artifact corrected peak current obtained using a given condition is devided by the artifact corrected reference peak current recorded within the same well.
    b. We used the third repetition of the reference peak current (sweep 4, Figure 6B) and the second repetition using the given condition (sweep 6); for the pH gradient we took the first repetition instead (sweep 5).
    c. For the normalization process, we considered error propagation.
7. **Averaging across sensors**
    a. Finally, we averaged across the artifact corrected, normalized net TMEM175 currents obtained for the same condition, again considering error propagation.
    b. In experiments with larger artifact currents, we neglected the standard error of the mean from averaging, which was typically lower or in the same range. Depending on the type of assay, errors from artifact variations or from TMEM175 current variations across sensors are higher.
8. **Plotting and Fitting**
    a. Artifact corrected, normalized and averaged data points were plotted and subsequently analyzed using distinct fitting equations as outlined in each figure description.

Variations for the ion selectivity assay

For the ion selectivity (Figure 2), we presented data recorded with the SURFE^{2}R N1 and the SURFE^{2}R 96SE. Using the SURFE^{2}R 96SE, artifact currents were more prominent relative to the currents recorded with the TMEM175 sample (Figure 2E), due to the lower sample amount used. Hence, we applied the same analysis procedure as outlined above, but used N=16 sensors per condition.

Using the SURFE^{2}R N1, we applied ten-fold higher sample concentrations to each sensor, essentially saturating the sensor surface, leading to higher signal-to-noise and low standard deviations of recorded peak currents across sensors. Additionally, currents recored with the control sample were ~20 fold lower compared to currents recorded with the TMEM175 sample. Therefore, we only show the mean and SD between sensors, without normalization or artifact correction (Figure 2D).

Variations for the K^+ concentration sequence

For the K^+ concentration dependence (Figure 3), we did not perform the in-well normalization, which was not required due to the high homogeniety of the reference control signals across the 96-sensor well-plate.

Variations for the H^+ flux assay

H^+ flux datasets (Figure 5) have been analyzed similarly as outlined above. Here, the SURFE^{2}R N1 was used and instead of 96-sensor well-plates, N=4 single sensors were used for both, control and TMEM samples. Additionally, all different H^+ concentrations have been measured sequentially on the same sensor.

Variations for the compound assays

For the analysis of compound data (Figures 6, 8), we chose a HTS suitable approach for data analysis. Error bars shown for compound data reflect the SEM obtained from automated data analysis using DataControl96, based on variations of the currents obtained with the TMEM175 sample.

SSME data shown in Figure 8 was not corrected for artifact currents, nor for off-target effects. We only tested the control sample for off-target responses applying the four highest concentrations used for each compound with N=1 sensor. We indicated the percentage of off-target effects relative to the

current recored using the TEMEM175 sample within Figure 8, as explained in the main text (section 2.9.2).

For the tool compounds (Figure 6), we subtracted the average artifact current in absence of the compound (-1.29 ± 0.52 nA, Figure 1K), before averaging to better capture a full block using 4-AP and zinc. We did not consider off-target effects, i.e. alterations of the artifact currents in the presence of compound, which were analyzed individually using the SURFE^{2}R N1 (Figure 9F) and found to be negligible for all compounds, but DCPIB.

Figure S1. Western Blot

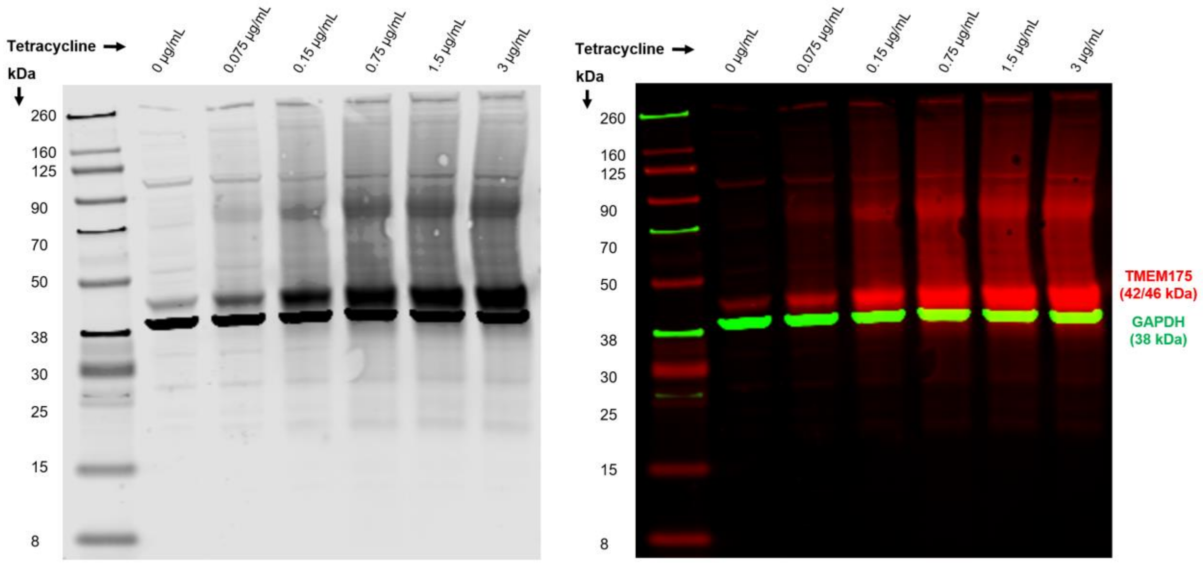

Tetracycline concentration dependent overexpression of TMEM175. As a control an antibody against Glycerinaldehyd-3-phosphat-Dehydrogenase (GAPDH) was used. Details are provided in the Materials and Methods section.

Figure S2. Ion selectivity of TMEM175 recorded with the SURFE^{2}R 96SE

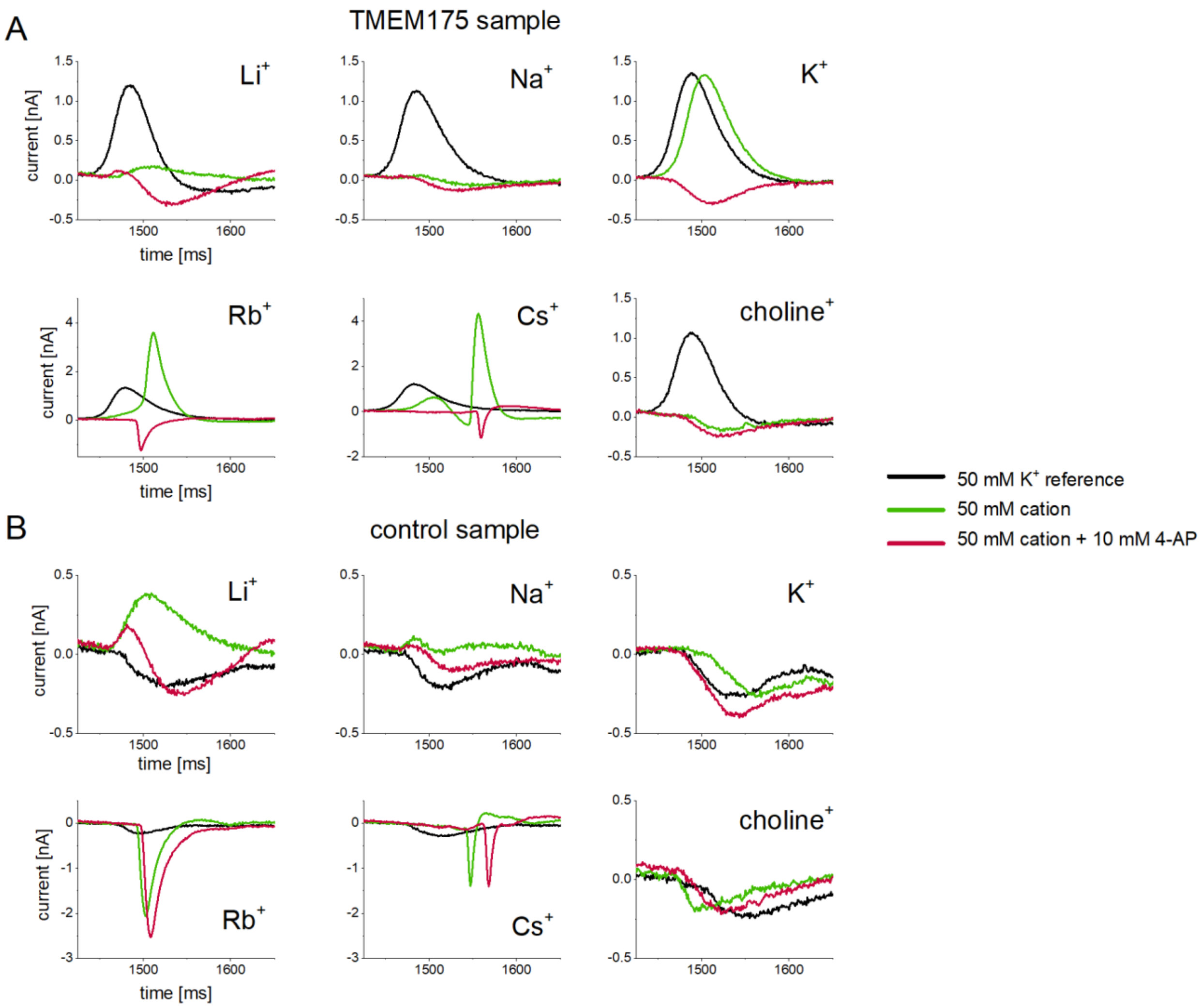

Ion selectivity of TMEM175 recorded on the SURFE^{2}R 96SE. Representative current traces recorded using different cation species on TMEM175 and control samples using the SURFE^{2}R 96SE. SEM and average peak current are shown in Figure 2E and 2F in the main manuscript. All traces in the same graph are recorded from the same sensor well in the following sequence: first, 50 mM K^+ is applied to record a reference current enabling in-well normalization (black traces); second, 50 mM of the indicated cation is applied generating currents reflecting the flux of the indicated cation (green traces); then the sensor is incubated for 3 minutes in NMDG-Cl containing solution supplemented with 10 mM 4-AP, followed by the application of 50 mM of the indicated cation in presence of 10 mM 4-AP. Between measurements sensors are washed with 50 mM NMDG-Cl.

Figure S3. pH dependence of K^+ flux through TMEM175 recorded with the SURFE^{2}R N1

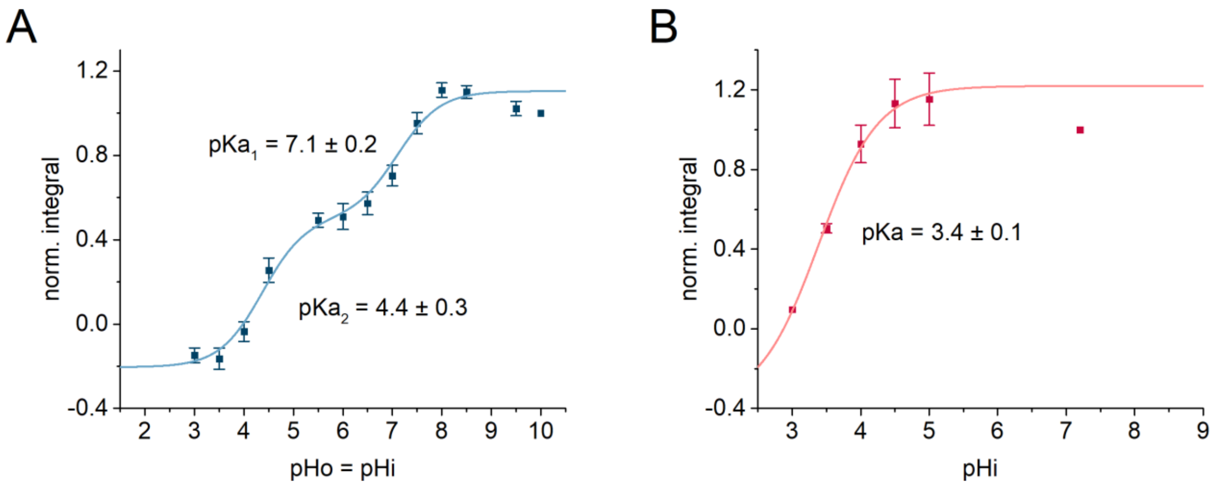

pH dependence of K^+ flux through TMEM175 recorded on the SURFE^{2}R N1. All pH values were recorded on the same sensor. Datasets were normalized to the value recorded at pH 7.5, before averaging. SD and average charge translocation from N=5 sensors are shown. The negative values observed in the charge translocation at very acidic pH values represent the artifact fraction of the TMEM175 sample current, as responses from the control sample were not subtracted. Using symmetrical pH conditions (A) and a pH gradient across the lysosomal membrane (B), we precisely reproduced all pKa values found with the SURFE^{2}R 96SE (Figure 4D, 4G).

Figure S4. HTS compatible workflow using the SURFE^{2}R 96SE

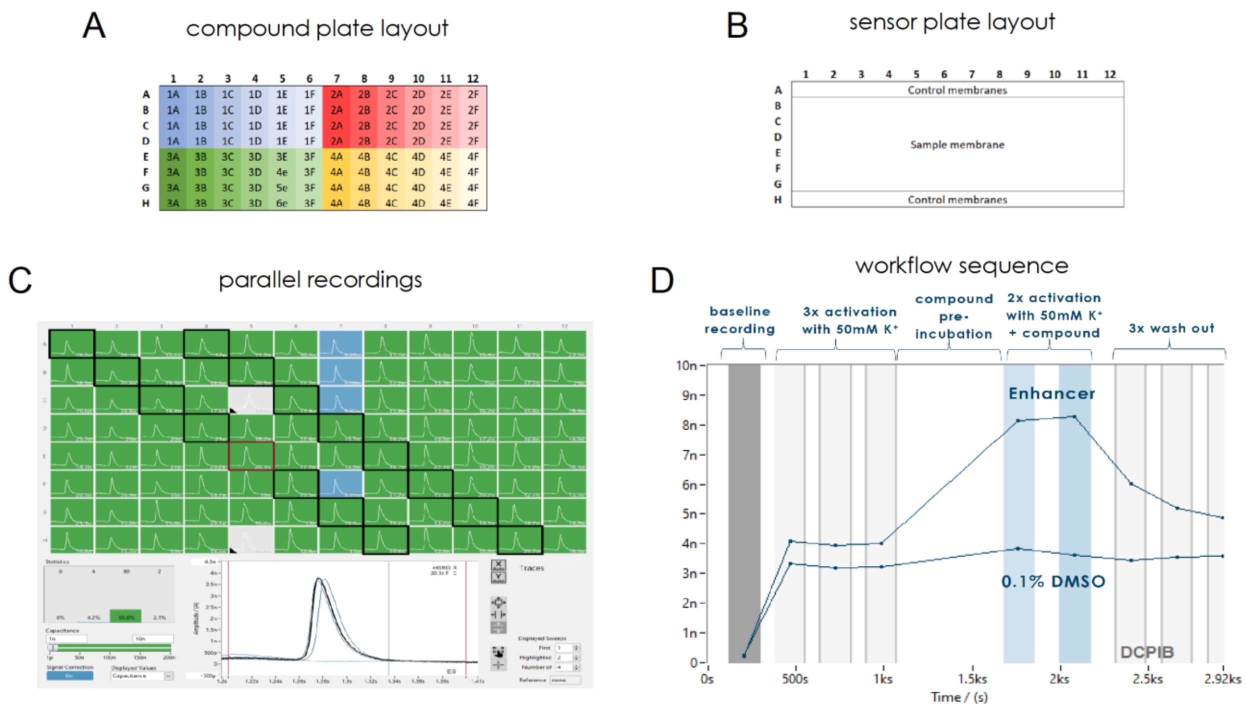

Example of a workflow for determining compound potency in an SSME screening assay. One 96-sensor well-plate is prepared with TMEM175 sample in rows B to G, while rows A and H contain the control sample. For the 96-sensor well-plates containing compound solutions, we selected a layout with 4x12 different compounds in the top half (wells X1-X4) and 4x12 different compounds in the bottom half (wells X5-X8) of the plate. This supports triplicate measurements on the TMEM175 sample and one measurement on the control sample per compound, with 24 compounds per plate. Parallel measurements of control samples allow for identification of non-specific compound effects and are specifically useful when higher compound concentrations above 50 µM are used. Alternatively – instead of measurements on 24 different compounds – IC$_{50}$ or EC$_{50}$ values for 4 different compounds using 6 different compound concentrations may be determined.

Independent of the type of compound measurement, each sensor will be treated with the same sequence of measurements and sensor washes. In SSME, compound experiments always follow a 3-step process: 1. activation, 2. comnpound addition and sensor incubation, and 3. activation in the presence of compound. Our typical workflow included the following steps.

Before the first, and in between measurements sensors are washed with non-activating solution. The first measurement is performed during injection of non-activating solution to record the baseline current, followed by three measurements exchanging non-activating for activating solution to determine the 'reference' current amplitude and test for signal stability within each single sensor. This measurement represents a full activation of the target protein, detecting a transport current in absence of the compound. Usually, we use the third measurement of each sensor as the reference ('100% signal'), which is used for normalization. For TMEM175 we applied a solution exchange from the non-activating solution containing 50 mM Na^+ to the activating solution containing 50 mM K^+.

After determining the reference signal, the compound is applied by rinsing the sensor with non-activating solution containing the compound at the desired concentration, followed by an incubation

time of 5 to 15 minutes, that may be required to equilibrate hydrophobic compounds. The data shown in Figures 6 and 8 was generated with an incubation time of 15 minutes.

Hereafter, the solution exchange measurement is repeated, exchanging the same non-activating, and activating solutions over the sensor, but this time both solutions contain the compound at the desired concentration. The signal may be affected by the presence of compound, either enhanced or inhibited, depending on the effect of the compound. The measurement in the presence of the compound is performed in duplicates and usually the second measurement is used for analysis, when compound equilibration is completed.

For each well, the percentage of inhibition and enhancement can now be calculated via normalization to the reference measurement. However, before normalization, artifact currents and non-specific effects of the compound on the control sample may be subtracted, if visible (compare Supplementary Methods). To generate the dataset shown in Figure 6, we subtracted the same average artifact current from all recordings with the TMEM175 sample before normalization, since we did not see large compound effects using the control sample (Figure 9F).

At the end of the measurement sequence the compound may be washed out of the sensor by rinsing with non-activating solution, and the target protein may be measured again via solution exchange in absence of compound to test for signal recovery. However, not all compounds may be successfully washed off the sensor. Signal recovery is not or only partially obtained for hydrophobic compounds that intercalate into the membranes. The complete workflow as outlined above takes 50 minutes, which is ~2 minutes per compound, with N=3 data points and one control measurement.

Alternatively, for a measurement sequence with increased throughput, there is a decent potential to simplify the workflow for a reduced measurement time of only 20 minutes for 24 compounds. The essential steps of the workflow include 1. sensor rinse using non-activating solution 2. duplicate activation using 50 mM K^+, 3. compound addition and ~5 minutes incubation, and 4. single activation in presence of compound.

Figure S5. Off-target compound effects recorded with the SURFE²R 96SE

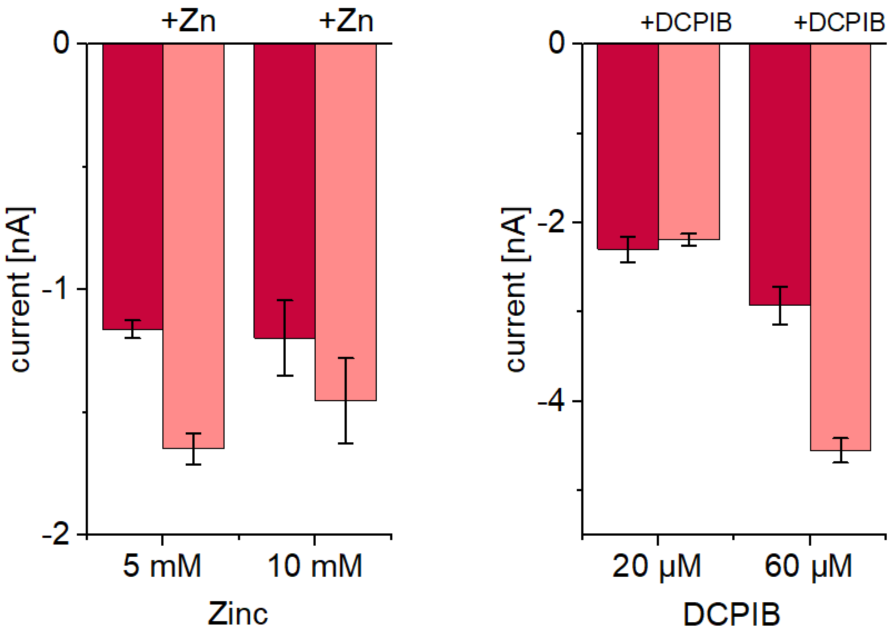

Currents recorded on control lysosomes expressing endogenous TMEM175 using the SURFE²R 96SE, in the absence and the presence of zinc and DCPIB. The controls were performed using the same sensor plate that was used for IC_{50} and EC_{50} determination shown in Figure 6 (in-plate control). A solution exchange from 50 mM Na^+ to 50 mM K^+ was used to record the peak currents. Dark red bars show averaged peak currents before compound addition; light red bars show the average peak currents of the same sensors in the presence of the indicated compound concentration. Equivalent recordings on the SURFE²R N1 are shown in Figure 9F. The results for zinc matched with those obtained on the SURFE²R N1, showing a small off-target inhibition. However, when adding 60 µM DCPIB, we observe that the current recorded with control lysosomes changes in the opposite direction compared to recordings on the SURFE²R N1, while in terms of magnitude there is a similar effect. Using the SURFE²R 96SE, the current decreases in presence of DCPIB, indicating an off-target inhibition, instead of off-target potentiation as found with the SURFE²R N1 (Figure 9F). DCPIB concentrations of 20 µM and below did not have an effect on the recorded control currents. The different effects may be due to different solution exchange properties and the higher time resolution of the SURFE²R 96SE when using 3 mm sensors (Bazzone et al. 2017a). Investigating the current traces recorded on the SURFE²R 96SE in presence of DCPIB, two distinct artifacts can be dissected: first, the fast decaying artifact current of negative amplitude, which is also present in absence of DCPIB and further decreased to more negative values when DCPIB is applied. Second, in the presence of DCPIB another slowly decaying artifact of positive amplitude is following. In the SURFE²R N1 recordings and due the lower time resolution, only the artifact of positive amplitude is observed and fully overlays the artifact with negative amplitude.

Figure S6. Stability of electrophysiological recordings with the SURFE²R 96SE

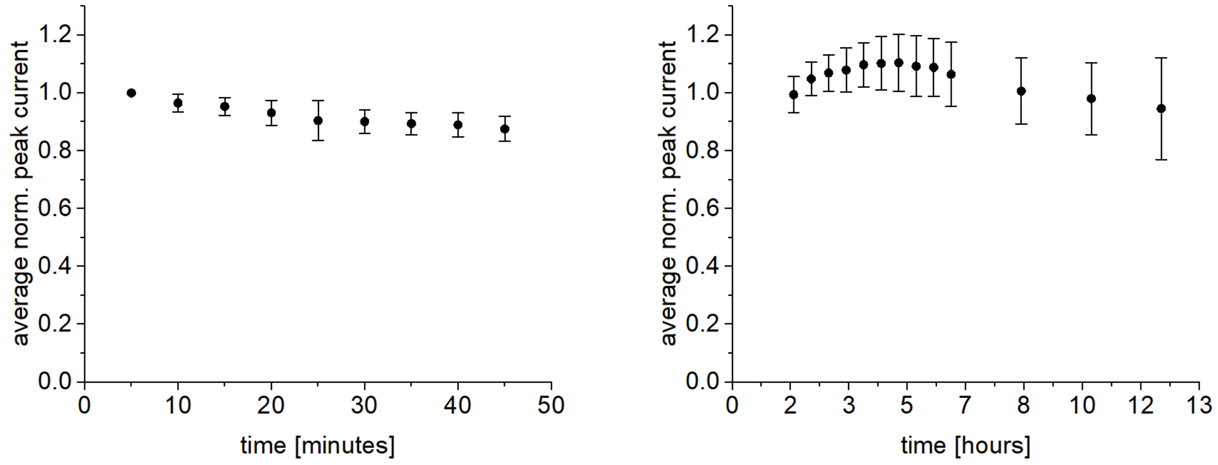

Stability of peak currents recorded on the SURFE²R 96SE over a time period of 12 hours. SD and average across N=96 sensors are shown. The peak current reflects initial glutamate transport rates of EAAT3, recorded on plasma membrane vesicles purified from EAAT3 overexpressing HEK293 cells. Compared to the TMEM175 assay, this assay is more complex because it requires the re-establishment of multiple ion gradients between individual measurements, and these gradients must remain stable over time. This includes an outward directed K^+ gradient and an inward directed Na^+ gradient. All measurement solutions contained 30 mM HEPES, pH 7.4 (NMDG) and 2 mM $MgCl_2$; the nonactivating solution contained 140 mM NaCl; the activating solution contained 140 mM NaCl and 1 mM L-glutamate; the resting solution contained 140 mM KCl.
